# Seasonal and Lunar Variation in the Emergence Time of a Population of *Uca lactea annulipes* (Milne-Edwards, 1837) at a Shore in Kuwait

**DOI:** 10.3109/01677063.2012.669439

**Published:** 2012-04-10

**Authors:** Layla Isa Al-Musawi, Edgar Wagner

**Affiliations:** 1 Regional Organization for the Protection of Marine Environment (ROPME), Kuwait; 2 Albert-Ludwig Universität, Institut für Biologie II, Freiburg im Breisgau, Germany

**Keywords:** Circadian rhythm, Ecological significance, Endogenous rhythm, Fiddler crab, Moon, Nonphotic zeitgebers, Ocypodedae, Tidal rhythm

## Abstract

This study monitored the endogenous emergence time of the fiddler crab *Uca lactea annulipes* (Milne-Edwards, 1837) in the field, for the first time, at an intertidal shore in Kuwait, from 1997 to 2001. The results revealed a significant cyclic change in the median emergence time as the season progressed from winter, through spring and summer, to autumn (.44,1.29,3.12, and 1.1 h prior to the dead-low tide, respectively). The data also revealed a significant shift in the median emergence time according to moon phase (2.27 h at new moon versus 2.56 h at full moon prior to the dead-low tide). (Author correspondence: l.almusawi@ropme.org)

## INTRODUCTION

The classical studies done on the circadian and tidal components of the color change, locomotor, and oxygen consumption rhythms of fiddler crabs in the 1940s and 1950s provided several important insights into fundamental biological rhythms (Brown, 1958; Brown & Webb, 1948; Brown et al., 1953; Fingerman, 1956; Hines 1953, 1954; Naylor 1958; Webb & Brown, 1959). The magnitude of the pioneering work established the existence of endogenous rhythmicity (Brown & Webb, 1948; Webb & Brown, 1959), the relative insensi-tivity of the period length to different constant temperatures (Brown, 1958; Brown et al., 1954; Hines, 1953, 1954), and the discovery of the tidal rhythm (Brown et al., 1953, 1956; Hines, 1953, 1954; Naylor, 1958). However, the vast majority of rhythmic studies carried out on these crabs were run under constant laboratory conditions in order to analyze the endogenous nature of their rhythmic behavior. The results of these studies were often noisy, with great variation between the individuals within a sample (Stephens, 1962), between samples of the same species from different localities, and between different species (Barnwell, 1966). These pioneering works tended to smooth out the high noise-to-signal ratio by pooling the responses of the tested animals. Hence, the smoothing out and the constant conditions of the laboratory together concealed important adaptive information embedded in this noise.

In the intertidal zone, biological rhythms related to the solar and lunar day compose a vital survival tool, which would adapt the animal to the cyclic changes in its habitat. A simple biochemical oscillator might suffice for this task, but it could not cope with the seasonal changes in many environmental cycles in the organism's habitat. In contrast, a circadian clock could accommodate these conditions, by being sensitive to external signals. Thus, the hands of the clock can be shifted forward or backward each day by light and other nonphotic zeitgebers, maintaining the clock's relevance to the environment of the organisms. The ecological importance of cyclic phenomena or rhythms for intertidal dwellers is quite obvious. There is a potential adaptive advantage of timing behavioral, metabolic, and developmental processes to the appropriate phase of the tide, night or day, and/or moon. As the overt circadian rhythms that can be observed in the physiology and behavior of animals are actually a cumulative product of the endogenous contribution from the circadian clock and exogenous masking from the environment, it is the environmental factors that fine-tune and synchronize the clock to local circumstances.

The correlation between the biology and behavior and the lunar-tidal cycle of intertidal dwellers has been long known to crustacean and molluscs gatherers. This empirical knowledge is reflected in the gathering practices handed down through the traditional knowledge of human communities in which such organisms form an important element in their cuisine and one main element of subsistence (Nishida et al., 2006a). These traditional gatherers not only realize the correlation between the lunar-tidal cycle of availability and distribution of such species in accordance to the lunar-tidal cycle, but they are also aware of its relation to the amount of flesh inside the hard shells of the targeted species and its effect on the female reproduction cycle. The ethnoecological study undertaken by Nishida et al. (2006b) utilizing such traditional knowledge documented that the biomass accumulation (meat) in some of the targeted mollusc increases during the spring tides and decreases during neap tides, reflecting the adaptive-ness of timing behavioral, metabolic, and developmental processes to the appropriate phase of the tide.

In the laboratory, the noise or the variation in the free-running patterns of the fiddler crabs species (Ocypode-dae) in constant conditions suggests that other signals from the environment entrain the clock while the crabs are inactive in their burrows during the high tide. The continuous entrainment of the endogenous component by different environmental factors may be the primary sources of the noise in data generated under the constant conditions of the laboratory. This study investigated two of the possible sources leading to such variations, by correlating the timed behavior of emergence to the surface as one aspect of the activity pattern of *Uca lactea annu-lipes* to the seasonal and lunar factors in the animals’ habitat, in an attempt to form a more detailed understanding of the possible sources of variation in such timed behaviors and the ecological significance of such variations.

## METHODS

### Study Site and Tidal Pattern

**FIGURE 1:**
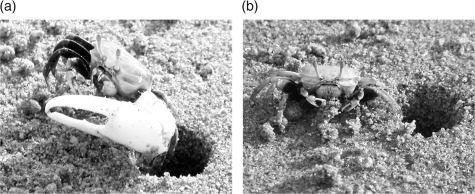
Sexually dimorphic male (a) and female (b) *Uca lactea annulipes.*

*Uca lactea annulipes* (Milne-Edwards, 1837) is a small Brachyuran crab (Figure 1), with a broad carapace and the abdomen being reduced and tightly flexed beneath the cephalothorax (Barnes, 1980; Crane, 1975). It inhabits the upper intertidal zone of shores with fine to muddy sand composition, and often lives in colonies (Jones, 1986).

The study site is an intertidal flat engulfing a shallow bay, protected from the south by a man-made pier and promenade in Kuwait City (N29°23′24′, E48°0′ll″) at Kuwait Towers. The beach is =290 m at its widest, and consists of a mixture of rocky, sandy, and muddy substrate (Tahmazian, 1998). The bay is of a lower elevation compared with the surrounding beach, and is the last to be uncovered by the receding tides, and the first to be filled with the incoming water.

The proper habitat of *Uca lactea annulipes* is exposed only during spring tides, when the tide amplitude drops to 1.3 m or less. In Kuwait, the shorelines experience two high and two low tides/d, at mean intervals of 12.4 h. The tidal amplitude varies on a given day with the season, between a minimum level of -.03 m and a maximum level of 3.96 m (as published in the Tide Tables by the Kuwait Ports Authority, State of Kuwait). Although the tidal pattern is semidiurnal, morning waters recede much further than afternoon ones in winter, and the pattern is reversed in summer. The time of the day when the appropriate amplitude of less than or equal to -1.3 m is reached varies between seasons. During the colder months of December through March, the tides with the appropriate amplitude occur only during one of the tides, and primarily the early morning until noon tides. Then, as the season progresses, such amplitudes occur during both tides, morning and late-afternoon tides. During July, the appropriate amplitude is reached only in the late-afternoon tides. Starting in August and lasting through November, the appropriate amplitude occurs during midday to late afternoon.

In order to control for seasonal and lunar factors, the sampling was randomized in the following fashion:

1. progressive sampling throughout the successive solar months;
2. progressive sampling at new and full moon phases; and
3. sampling twice on the same day when one low tide amplitude is lower and the other is higher than -1.3 m.

### Filming and Sampling Protocol

All observations were carried out during the daylight hours. The reported data were collected from March 1997 until August 2001. An area in the center of the population, with a mixture of sandy and muddy substrate, was defined as the focal study area. It consisted of 16 m^2^ in the upper intertidal zone, parallel to the shoreline. Then, a stratified design was devised to sample the population in accordance to the tideline. This was accomplished by further subdividing the study area into a grid of four equal transects parallel to the shoreline, and sloping upwards, to assure that samples were taken across the entire gradient of the slope.

Emergence from the burrow after the recession of the water was selected as the main parameter of a timed behavior. It is an extremely obvious behavior. Prior to emergence, the surface of the sand bulges; then, the animal tosses away the sand with the tip of its walking legs and emerges to the surface. The fact that individual crabs live in separate burrows facilitates quantifying this timed behavior, while avoiding any duplication in counts.

The water starts receding roughly 3.5 h before the dead-low tide, which is defined as time 0 (T0), and the beach is uncovered for another 3 to 3.5 h. In order to standardize the data, the time of emergence was related to the time of the dead-low tide, designated as T0. The absolute emergence time was calculated by subtracting the emergence time from the time of the low tide. The timing of emergence was recorded using three video cameras (Panasonic RX18c; Japan). The cameras were always mounted vertically at fixed points of the sampled transect. Each camera filmed an area of .5 x .5 m^2^ across the slope. In order to enhance the sample size, counts were made by the observer seated 1 m away behind a blind, noting the time of emergence and the position of the animals on the stratified grid. This method of sampling increases the sample by 3-fold, overcomes the limitation of direct observation, and reduces disturbance to the animals. The sampling method was selected and developed to minimize any interference with the natural setting of the studied species and to avoid destruction of its proper habitat. Thus, the sampling protocol conforms to the international ethical standards as set forth in Portaluppi et al. (2010).

### Statistical Analysis

Nonparametric statistics were used, due to the repeated sampling of the same population, and to avoid making any assumptions about normality, homogeneity of variance, or the precise form of the underlying distribution. To detect changes in the median timing of emergence as a function of the ambient temperature as the season progresses, the data were first tested using a distribution-free analysis of variance (ANOVA) with multiple comparison based on Friedman two-way ANOVA (Siegel & Castellan, 1988) to confirm the hypothesis of no treatment differences between each solar month in the different years. Then, the data of the corresponding months were treated as one block, and the pooled data were tested at the following levels:

1. Correlation between the timing of emergence and ambient temperature. At this level of investigation, the data were analyzed using the Kruskal-Wallis rank coefficient regression analysis (Siegel & Castellan, 1988) to determine the correlation between the timing of emergence and ambient temperature.
2. Seasonal emergence patterns. The shift in the median of the different solar months was tested using a distribution-free ANOVA and multiple comparison based on Friedman two-way ANOVA (Siegel & Castellan, 1988) to detect seasonal variation.
3. Lunar variation in the timing of emergence. The data were further analyzed as a function of moon phase. The new moon phase was defined as d 1-4 of the lunar month, with a total of 109 d of the research period that fit this definition. The full moon phase was defined as phase d 13-16 of the lunar month, with a total of 102 d of the research period. At this level of investigation, the Mann-Whitney test of median (Siegel & Castellan, 1988) was applied to the collective data to detect variation in the timing of emergence as a function of the lunar phase.

## RESULTS

### Correlation Between the Timing of Emergence and Ambient Temperature

The timing of emergence shifted as the season progressed from winter to summer and as the ambient temperature increased. As the temperature increased, the animals become active above the surface sooner (Kruskal-Wallis rank coefficient, *p*< .001).

### Seasonal Emergence Patterns

**FIGURE 2:**
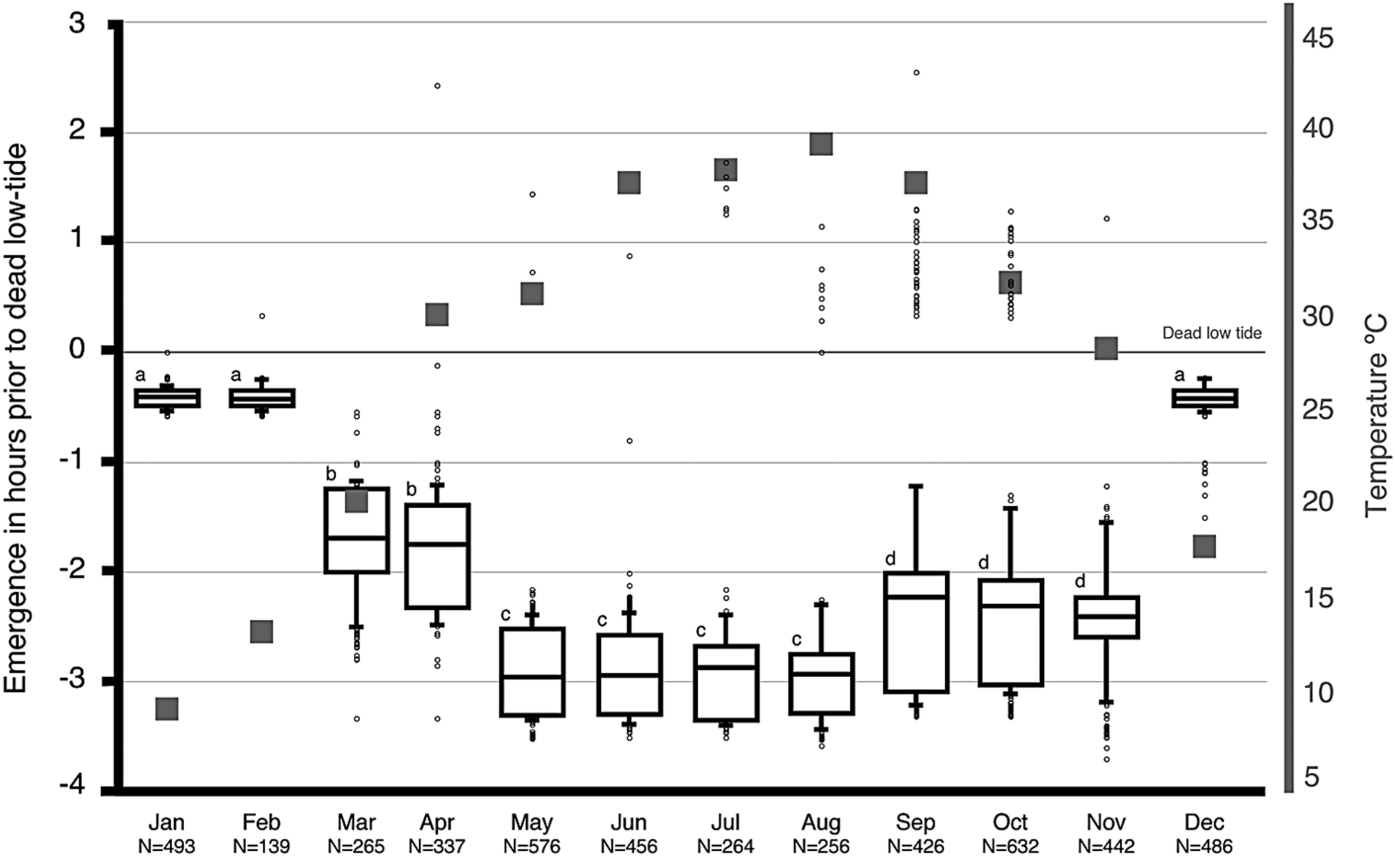
Seasonal variation in the tidal rhythm of emergence. The graph summarizes the emergence data collected in the years 1997-2001. The x-axis is divided into the 12 mo of the solar year, whereas the y-axis represents the time (in h) before and after the dead low tide (the horizontal line at zero denotes the time of low tide). The collective data of a given month are presented as a box plot. This graphical method displays the full data range of the given variable, including outliers drawn as small circles. The horizontal line in the middle of the box represents the median 50% value. The number of the different emergences (N) observed is indicated below each time interval. A lower case letter is given to groups with statistically equal medians. Squares are drawn from the average low temperature of the day, as supplied by the Climatological Division, Meteorological Department, Directorate of Civil Aviation, State of Kuwait.

Figure 2 summarizes the emergence data collected in the years 1997-2001. The plotted data suggest a gradual change in the median emergence time as the seasons progress, from winter to autumn through spring and summer. This evident shift in median emergence time is highly significant (Friedman two-way ANOVA, *p* <.0001).

#### Winter Emergence Pattern

On days when the ambient temperature was 10°C or less and surface temperature 8°C or less, no animals emerged (Mann-Whitney test of variance, p<.001). During the cooler months of the year (December-February), the animals delay their on-surface activity. No significant difference was detected between these 3 mo (Friedman two-way ANOVA, *p* > .05), and the median emergence time was .44 h prior to T0.

#### Spring Emergence Pattern

The spring season is a rather short season in Kuwait; it consists mainly of March and April, and as the temperature starts to rise in these 2 mo, the animals become active earlier than in the winter months. There was no significant variation in the median emergence time between these 2 mo (Friedman two-way ANOVA, *p >.* 05), and the median emergence time was 1.43 h prior to T0.

#### Summer Emergence Pattern

A further increase in the median emergence time is seen during the long summer season (May-August). As the ambient temperature further increases from May through August to reach its maximum, the median emergence time increases to 3.01 h prior to T0. There was no significant variation in the median emergence time between these 4 mo (Friedman two-way ANOVA, *p* > .05).

#### Autumn Emergence Pattern

A gradual decrease is evident as the temperature starts to cool down once again during September and November. There was no significant variation in the median emergence time between these 3 mo (Friedman two-way ANOVA, *p >* .05), and the median emergence time was 2.05 h prior to T0.

### Lunar Variation in the Timing of Emergence

**FIGURE 3:**
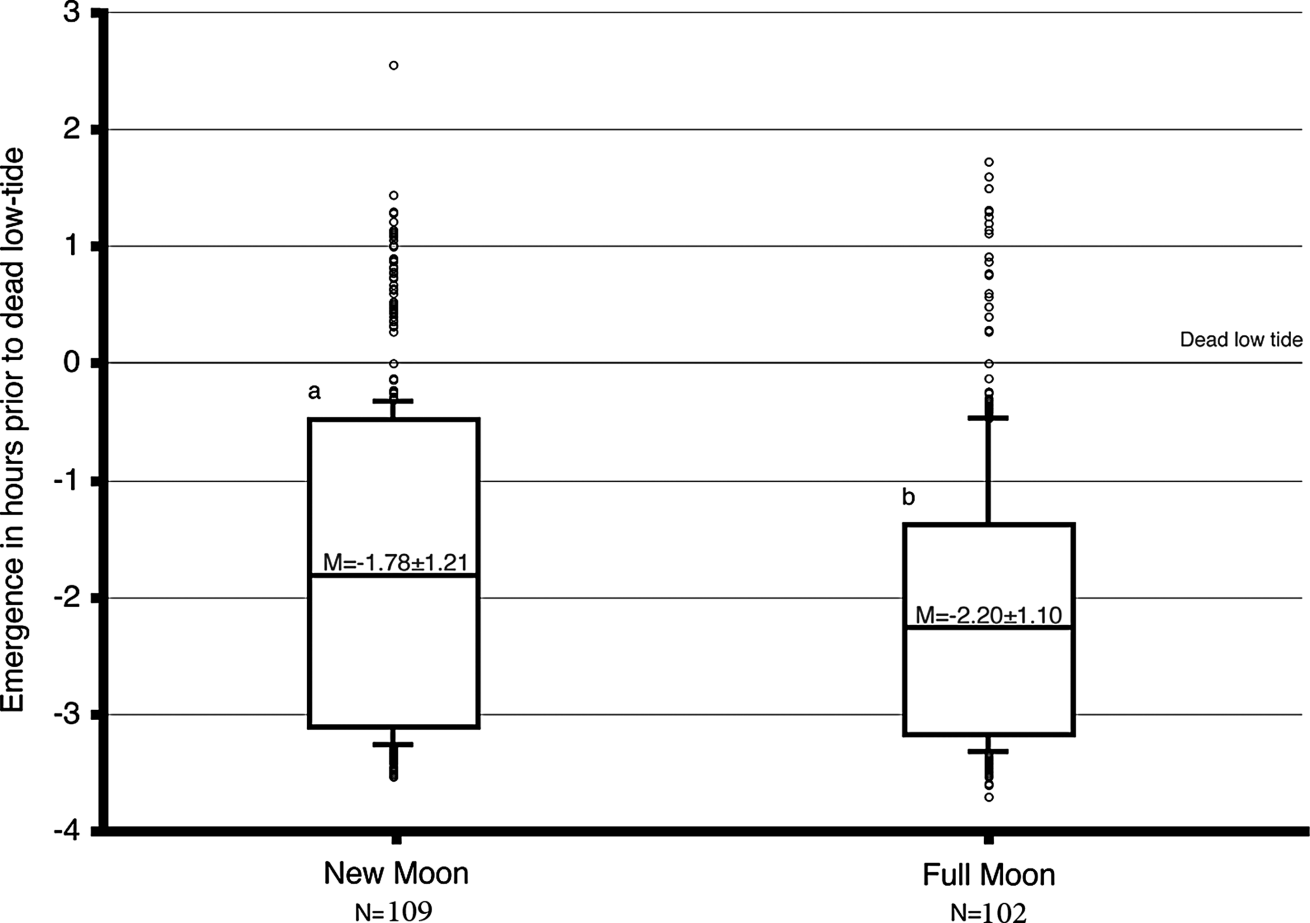
Lunar variation in the tidal rhythm of emergence. The graph summarizes emergence data as a function of the moon phase. The collective data of a given phase are presented as a box plot. This graphical method displays the full data range of the given variable including outliers drawn as small circles. The horizontal line in the middle of the box represents the median 50% value. A lower case letter is given to groups with statistically equal medians. The number of the different emergences (N) observed is indicated below each time interval. A lower case letter is given to groups with statistically equal medians.

The distribution of the emergence time as a function of lunar phase is illustrated in Figure 3. At new moon, the median emergence time is 2.27 h prior to low tide (T0), and the median emergence time around full moon equals 2.56 h prior to T0. The shift in the median is highly significant (Mann-Whitney test of variance, *p* < .001). Around the full moon phase, the deviation of the timing of emergence is constrained, and the animals are more synchronized, whereas the range of emergence time around the new moon is more relaxed (Figure 3).

## DISCUSSION

*Uca lactea annulipes* are intertidal dwellers, with each individual living in a single permanent burrow in the intertidal zone. Their proper habitat is uncovered only for a few hours each day around the new and full moon, during spring tides. This small window of time represents their chance of survival, the time when the animals could come to the surface to feed, repair their burrows, and court mates. The endogenous nature of a rhythmic behavior enables these organisms to be active during the appropriate time window. Should they miss this time window, they would have to survive until the next cycle. Therefore, their clocks should be synchronized by external signals to accommodate the complex environmental conditions in which they live, and to be fine-tuned to particular conditions in their habitat that may vary from one locality to another.

Light has been long established as one of the main universal zeitgebers, and phase-resetting by light has been extensively studied. However, for organisms in the complex intertidal habitat, one zeitgeber might not be sufficient to synchronize all their life cycle functions (Warman & Naylor, 1995). Zeitgebers, such as hydrostatic pressure, temperature, and microgravitation, seem to have profound effects on setting or phasing the clocks of these organisms.

The results of this study display the close relation between the timing of emergence and low tides, and show that these animals exhibit a tidal rhythm throughout the year. Yet, the annual pattern of synchrony of the studied behavior is not perfectly parallel to the predicted changes in tides and the resulting periodic change in the hydrostatic pressure of the receding tide. However, these field data cannot exclude a potential role of the changing hydrostatic pressure in entraining the endogenous rhythm in the adult crabs, generally, and the timing of emergence, in particular. On the other hand, the change in the overt rhythmic activity of the crabs in response to the change in the ambient temperature due to the progression of the seasons suggests that temperature is a major factor in the entrainment of endogenous clocks, and that diel variation may be one of the possible zeitgebers entraining the rhythm in these organisms. The naturally occurring changes in ambient temperature, and subsequently the substratum, mimic the effect of light and dark cycles, and can reset the clock and synchronize the animals to the particular changes in their habitat.

The change in the response of the crabs is not only evident in response to changes in the seasons, but also the variation in the response is affected by the range of change in the ambient temperature. The proper tides in the winter months occur early in the morning. In such days when the temperature was below 10°C as the water uncovered the intertidal zone, the crabs remained inactive and did not emerge to the surface until much later when the ambient temperature increased above 10°C. The seasonal effect is further evident in the correlation between the response of the animals to the degree of variation in the ambient temperature. As the temperature rises in the spring months of March and April, with a mean temperature of 20°C and 30°C, respectively, the variation of about 10°C did not result in significant variation in emergence time. Whereas the emergence time of the crabs in the extremely arid summer months, May through August, with mean ambient temperature rising above 30°C from May and reaching to 40°C in August, did not significantly vary in response to a variation of 5°C between May and June through August.

The results of this study are in agreement with the conclusion of recent laboratory investigations on other organisms. Liu et al. (1998) presented evidence that temperature changes can reset circadian oscillators of the mold *Neurospora,* and act as strong zeitgebers. They found that temperature changes, especially increase in ambient temperature, lead to posttranscrip-tional changes in the level of *frq* mRNA and FRQ protein, which are essential for the *Neurospora* clock. At elevated temperature, the FRQ protein oscillated around higher levels, and at colder temperature around lower levels. Also, the response to the changes in temperature was rapid, such as in response to changes in light. However, unlike light, the resetting by temperature in the above study appears to be the result of changes brought about directly within the oscillator. The level of FRQ protein is adjusted in response to the alteration in ambient temperature, and, hence, resetting the circadian cycle by changing the set point and internal dynamics of the feedback loop.

The results of the current field study imply that the rhythm of the crabs may very well be entrained by changes in ambient temperatures. The progression of seasons would shift the organism from one temperature level to another, and phase shift the clock to the local environmental factors. The changes in temperature affect the phase of the rhythm; so, in summer, the crabs emerge soon after the recession of the tide, and as the temperature drops in autumn and winter, the phase of the rhythm is adjusted accordingly. Also, the results of this study suggest possible entrainment by mi-crogravitational changes. It is generally believed that *Uca lactea annulipes* is a diurnal species, and, hence, not subjected to the effect of the prolonged light cycle around full moon. Nonetheless, the animals seem to become active earlier on days with full moon nights, and the emergence time is more synchronized around these days. These results suggest crabs are sensitive to the microgravita-tional changes through the lunar cycle, and may indicate possible pathways of detecting changes in microgravi-taional forces by these animals, like many other organisms that exhibit rhythmic behaviors or phenomena that correlate with the change in the Earth's gravitational force due to the lunar cycle (Zürcher et al., 1998). Unlike light, changes in temperature and microgravitational forces could be sensed by the crabs even when they are deep in their burrows and covered by the tide. If the crabs are entrained by such environmental signals, and such mechanisms of entraining the endogenous rhythm partakes in the physiology of these crabs, it would be of fundamental advantage to these animals, as it would render them active around optimum conditions, and ultimately enhances their fitness.

Reproduction of many intertidal dwellers is thought to be adjusted to an optimum time of the year and lunar phase. Timing the breeding cycle to coincide with the warmer months of the year ensures releasing the larvae in favorable temperatures, and when sufficient nutrients are available to both adults and larvae (Robertson, 1991). Moreover, there is evidence that crabs of the genus *Uca* mate preferentially around the full moon and release their larvae around the new moon (Greenspan, 1982). This semilunar rhythm of reproductive activity is also reported for many intertidal organisms (Hauenschild, 1960; Korringa, 1947). Spacing reproductive cycles in accordance with the lunar phase restricts reproduction to a few days in the lunar cycle, and, hence, ensures synchronization of the mating cycles of mature individuals (Hauenschild, 1960; Korringa, 1947; Mizushima et al., 2000; Morgan, 1987; Morgan & Christy, 1994, 1995). Furthermore, timing the release of larvae during spring tides around new moon is advantageous to both females and larvae, as the darkness would reduce predator risks to adult female and permit the released larvae to escape predators. Also, the faster current during spring tides ensures maximum dispersal (Robertson, 1991).

In conclusion, noise in data generated in the constant condition of the lab could have arisen from not thoroughly accounting for the animals’ natural zeitgebers as well as from the sampling protocol. Data from animals drawn from different season or lunar phase should be analyzed separately, and measures should be taken to compensate for individual deviation. Animals synchronized to a particular locality and/or season will exhibit different phases of synchrony, which would override laboratory conditions and affect the results. Laboratory studies and data analysis methods should pay more attention when analyzing data from constant-condition experimental settings, to compensate for possible seasonal and lunar factors affecting the behavior of their test organisms.
